# A Methodology for Evaluating the Progression of Damage in a Glass Fibre Reinforced Polymer Laminate Subjected to Vertical Weight Drop Impacts

**DOI:** 10.3390/polym13132131

**Published:** 2021-06-29

**Authors:** Patrick Townsend, Juan C. Suárez-Bermejo, Álvaro Rodríguez-Ortíz

**Affiliations:** 1 Escuela Superior Politécnica del Litoral (ESPOL), Facultad de Ingeniería Marítima y Ciencias del Mar (FIMCBOR), Guayaquil 09-01-5863, Ecuador; 2 Universidad Politécnica de Madrid (UPM), Escuela Técnica Superior de Ingenieros Navales (ETSIN), 28040 Madrid, Spain; juancarlos.suarez@upm.es (J.C.S.-B.); alvaro.rodriguez@upm.es (Á.R.-O.)

**Keywords:** impact, GFRP, microcracks, curing time

## Abstract

This study describes a methodology that allows evaluating the behavior of a glass fibre reinforced polymer (GFRP) laminate impacted by a vertical weight drop, analyzing the damage that occurred inside. The purpose of the designers was, by means of characterization tests of the curing processes, evaluation of the cohesion of a particular laminate, application of vertical tests by weight drops and with the use of the readings of an accelerometer in a single direction, know the trend of how intralaminar breaks in the matrix and interlaminar breaks between layers occur. It is proposed to establish the behavior of the laminate before the tests by analyzing curing times, for after the tests by observations with penetrating fluorescent inks. This allows researchers to know the response of the laminate to the loads imposed on the applied structure. For the tests, prepreg material cured outside the autoclave in an oven was used and qualitative quantification of the damage by observing sections of the tested material infiltrated with penetrating fluorescent ink exposed to ultraviolet light.

## 1. Introduction

The use in industry of composite materials—in this case glass fibre reinforced polymer (GFRP)—is very varied, and includes the construction of panels, small boats, houses, structures and large fishing vessels. As it is a laminated material that is protected with paint and sealed in order to prevent moisture from affecting it, it is very hard to determine its useful life according to the work it performs. The micropores and microcracks that are formed inside are not easily detectable until when the laminated panel has gained a lot of flexibility and this change is visible to the eye. The energy imposed on a GFRP panel is distributed between the matrix and the reinforcing fibers, causing great fatigue damage. Sometimes very light impacts may already cause damage to the matrix.

At present, improvements are being made in the performance of these materials. The invention of prepregs, which are GFRPs that come in ready-to-cure sheets, with the inert resin injected at the factory displaying almost zero percent porosity, has improved the performance of GFRPs. For shipbuilding, due to the issue of lowering production costs and taking advantage of the high qualities of prepregs, manufacturers have developed OoA or “cured out of the autoclave” type, which require a vacuum bag pressure procedure after lamination and a drying process in an oven or stove for the time recommended by the manufacturer. This type of process has made GFRP more affordable for use in the manufacture of all types of structures.

The structure of a laminated composite material is related to the dissipation of energy in its stacked layers [1]. This is the tricky thing about GFRPs, since the energy that is dissipated in the material after impact is not distributed uniformly, but rather the orthotropy of the material allows any impacts imposed on the material to cause it to have different behaviors within of the structure. The stresses and strains on the laminate are not uniform and vary in the direction of the laminate surface. Transversally, the stresses jump between layers and depending on the type of compound and its orientation, the deformations make this type of damage unpredictable [2,3]. Within the layers, the damage caused by pressure during impact phenomena in the first instance causes the matrix to break randomly. Then it produces delamination between layers, and as the deformation of the panel increases, these join with the cracks in the matrix and form continuous steps.

Many types of tests are carried out based on the principle of conservation of energy, in which an impactor device is launched from a height above the sample to be studied, falling freely with an acceleration value equal to gravity (g). According to research by Lopes and Seresta [4], this technique provides very complete results based on the observation that all potential energy is converted into kinetic energy and this, when transferred to the test specimen under impact, produces damage that is transmitted on its surface. GFRP materials when subjected to this type of impact test dissipate the energy received through the laminate. Depending on its intensity, it turns into damage of different types.

The stages of damage produced by an impactor on composite material in GFRP laminates were observed by Choi [5,6], and then experimentally repeated by Ahmed [7]. At the moment of impact, during the initial stage, the panel begins to flex elastically, generating internal stresses and deformations as a result of the response to the different energies that are transferred from the impactor. After the elastic limit or material damage threshold has been exceeded, the laminate cannot return all the impact energy and the first intralaminar cracks begin to appear and, depending on the orientation of the fibers and the imposed stresses, the matrix breaks due to the deflection of the panel that generates tensile and compressive stresses. This is known as the second stage. The effects are generally seen in a cross-section, as marks normal to the laminate surface, which do not follow the same order jumping from layer to layer. If the impactor continues to act on the panel and introduces more energy into the laminate, it goes to the third stage, where interlayer delamination appears. This effect is oriented in the form of a ladder joining the cracks in the matrix. These delaminations generally surround the impact zone and correspond to the flexion pull zone. If the energy delivered by the impactor at the moment of impact continues to act on the deformation zone, there is a fourth stage in which the fibers break and begin to be visible from the outside. In this last stage it becomes very complex to quantify the type of damage.

The energy behavior during the impact has been studied using an accelerometer with a computer data acquisition system, obtaining good results for parameters such as force, displacement and returned energy, as demonstrated by Baucom [8] and Svenson [9]. These data are used to generate the characteristic curves of acceleration vs time and force vs energies that have been developed in multiple investigations of GFRP materials such as those presented by Elavenil [10] and Grasso [11]. From the data obtained by the accelerometer, they develop the analysis of the behavior of the laminate during impact.

The initial energy applied by the acceleration of the weight on the specimen is decomposed into stored or elastic energy, the viscoelastic energy that corresponds to the capacity of the material to dissipate the energy received during the impact and that propagates the damage, the energy returned, and the energy consumed associated with strain rate or kinetic energy. The force that produces the first delamination is known as the critical energy and it is related to the material damage threshold or delamination threshold. Impacts below the threshold are considered subcritical and those above the damage supercritical

To integrate all the information obtained experimentally and use it in the design of new structures, it is necessary to propose damage models that allow characterizing the behavior of the material. Review on this topic can be found in Liu and Zheng [12] and Zhou [13]. The failure modes that occur in materials are responsible for the modes in which delamination and loss of stiffness occur [14,15,16]. Delamination or separation of the individual layers is a major defect that does not allow a structurally sound laminate to be maintained. As the interlaminar toughness of laminates is the lowest hardness, a crack easily propagates under tensile stresses at the interface.

A full report reviewing the resistance to damage imposed by impact of a GFRP was published by Tomblin [17], from which Zilong [18] confirmed that the evaluation of residual resistance is an important complement in the investigation of a sequence of impacts from different heights, and allows one to obtain an exponential relationship between the energies that decreases with the compression force prior to the failure in the test, subjecting an impacted specimen to a pressure on the plane of the delamination to obtain a trend of how the impact has been changing the flexibility of GFRP.

Based on all the knowledge of the behavior of delamination within the GFRP, a methodology of observing the behavior of a prepreg laminate with nine layers is proposed, to establish a relationship between the damage, the energy imposed and the speed deformation during vertical impacts due to dropped weights.

Prepreg panels will be manufactured outside the autoclave, it will be verified if the curing process carried out was acceptable, they will be vertically impacted and then characterized with penetrating fluorescent inks for observation under ultraviolet light, and the results obtained using a one-way accelerometer. Thus will serve to establish the damage mechanism, the failure trends in the laminate, and the energies at which the laminate damage trends change.

## 2. Materials and Methods

**Figure 1:**
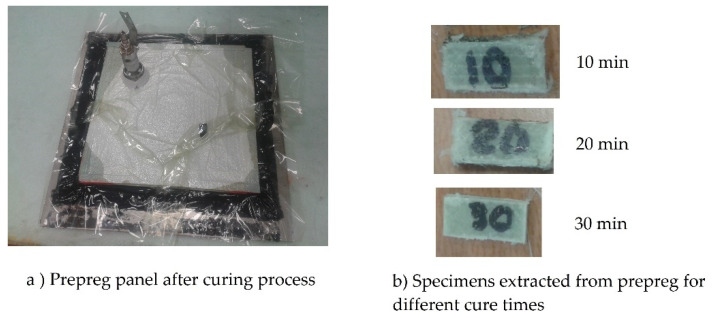
Test specimen manufacturing process: (**a**) Prepreg panel after curing process in vacuum pressure oven, (**b**) example of specimens drawn from prepreg for different cure times.

For the preparation of the test specimens, pre-impregnated material (prepreg) with nine layers has been used. The stacking sequence is [−45°/0°/+45°]_3_. Importantly, the prepreg fabric comes in three warps already wetted with resin [−45°/0°/+45°] in such a way that three warps must be stacked to make nine layers. Adhesion problems should be reviewed between warp group addtions. These were prepared on a Teflon tool for stacking. The selected material is for curing outside the autoclave, in an oven with vacuum pressure. Figure 1a shows a panel already cured outside the oven. The breather fabrics and corks used allowed it to breathe through the edges, eliminating gases and some resin. This should ensure that there are as few pores between the layers. The oven time ramp was 90 min according to the manufacturer’s instructions. For the type of analysis to be performed, it was necessary to verify the curing procedure over time. Therefore, three types of specimens were cured. The first only with a 10-min stay in the oven, the second with a 20-min stay in the oven and the third with a 30-min stay in the oven. The temperature controls should be set after the oven reaches 120 degrees Celsius, which corresponds to that given by the manufacturer for the curing process. The samples had to be cut into small pieces after being removed from the oven. Figure 1b shows the specimens ready for the stuffing machine. This procedure allows one to ignore errors due to lack of impregnation in the layers and to verify that there is a sufficient adhesion bond within the laminate layers.

The embedded equipment allows the specimens to be encapsulated to expose the cross-section of the laminate. Once embedded, a fine powder sanding system must be applied. This sanding exposes the inside of the laminate so that it can be viewed under a microscope. Generally between ×100 to ×300 is enough zoom to check the curing process. In the specimens placed in the plastic capsule the section of the laminate is exposed, allowing an acceptable observation that will evaluate the amount of pores and defects during curing to relate the interlaminar and intralaminar breaks that occur in the tests, differentiating those that correspond to impact damage or failure of the material response to defects.

**Figure 2:**
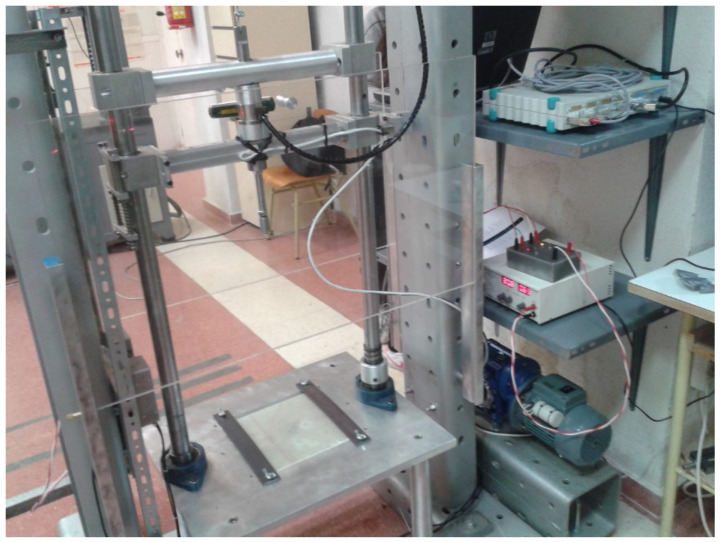
Weight drop vertical impact equipment manufactured exclusively for the developed experiments.

The equipment used to impact the panels is a vertical test by weight drop system, available at the Polytechnic University of Madrid, and has a one-way gravitometer reader that allows testing the deformation of the panel during impact. This equipment was manufactured exclusively for the type of tests carried out in the present investigation. For this it uses 30 × 30 cm^2^ panels and the heights at which it can be calibrated do not exceed the energy levels evaluated. This reader allows to record the acceleration changes of the impact sphere as it deforms the panel and breaks the matrix. The acceleration forces change until the deformation comes to a stop. As it is of the vertical type, the restitution is reflected as acceleration contrary to impact. Figure 2 shows the equipment in detail.

The data provided by the accelerometer, which corresponds to the G forces of impact, can be evaluated using the following speed formulation in order to relate the speed at which the damage occurs with the observation of the location of the damage to establish trends in the results. The calculation of the $v_{i}$ for each instant of the process is presented in Equation (1). $v_{i}$ is the velocity of the impactor in an instant of time i while pressing the laminate and deforming it, $v_{impacto}$ is the velocity of the impactor at the instant of impact on the panel surface, $G_{n}$ corresponds to the acceleration provided by the accelerometer in gravitational forces, t the reading time of the data provided by the equipment and n the valid number of data obtained according to the calibrated frequency for the equipment:

(1) $$v_{i}={\left\{v_{impacto}+\overset{i}{\underset{n=1}{\sum }}\frac{1}{2}G_{n}\left(\left(t_{n+1}-t_{n}\right)\right)\right\}|}_{i=1}^{n}$$

The impacted specimens will be immersed in fluorescent penetrating ink after making a hole with a 0.2 mm drill for the liquid to penetrate. These are immersed in the ink for a period of 2 min which has been considered as sufficient time for the ink to reach all the mined and damaged areas inside the material. GFRP compound. It should be noted that the regions in which the cut of the specimen has been damaged by the saw must be identified, and if these damages in any way affect the areas with delaminations. After this process, they are placed on absorbent powder in order to extract the remaining ink that did not penetrate. Finally, they are washed with plenty of water so that no dust or substances from the process remain on the surface. To expose their interior the specimens are cut with a diamond blade lubricated with oil so that the friction does not drag particles of the laminate into the microcracks, and this would provide a false reading during the observation. The cut sections are exposed to UV light and finally photographed for analysis.

**Figure 3:**
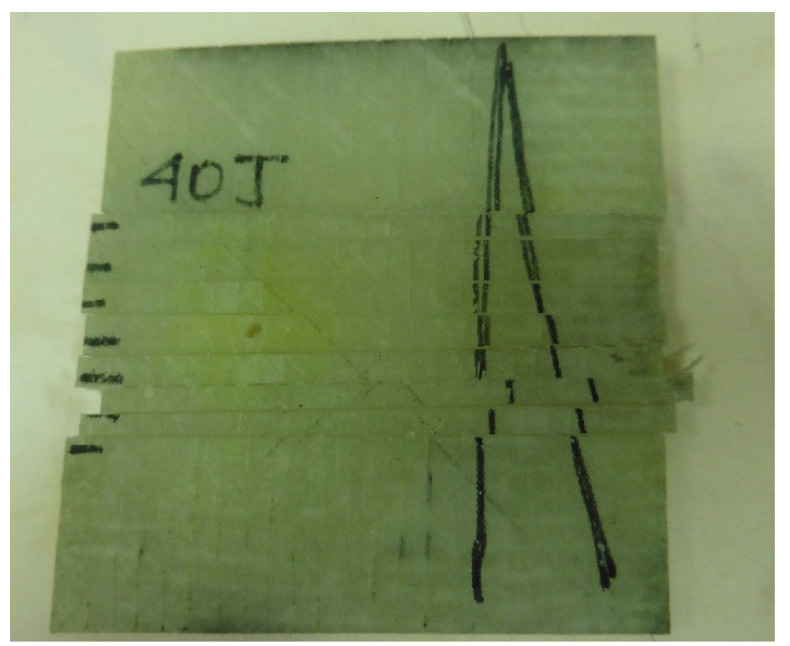
Section cuts for selection of exposure to fluorescent light for a specimen extracted from a panel impacted at 40 Joules.

Once the sections have been obtained, the accelerations delaminating the layers will be evaluated numerically, the interrelation with the breaks presented will be identified, and the deformation speed will be calculated in the states of breakage and restitution. This is for the purpose of evaluating the incidence of damage with the applied energies to understand the behavior of the laminate and detect any trends. Figure 3 shows an example of the section cut for a specimen tested by impact and that has been previously penetrated by fluorescent ink. It will also be important to photograph the section surfaces in zoom to locate pores in the final process and how these have been affected by the dragging of the matrix material during cutting. White light will be used for this. The matrix residues, being polymers crushed at the edges of the cut, will create a reflection of light around it, which will allow it to be fully identifiable. Cut sections that have a lot of material carryover will not be considered for analysis.

## 3. Results

The experimental results according to the developed tests are as follows.

### 3.1. Results on Curing Tests

Three panels were cured for just 10 min in the oven under vacuum pressure at a temperature of 120 degrees C. The oven used had a ramp of 15 min until reaching the curing temperature. In the case of the 20-min curing time of the three panels, one failed in the initial ramp in terms of its duration, and it was not considered to represent a good comparison. In the case of 30 min of curing, if it was possible to work the three panels and extract the samples. This is detailed in Table 1.

**Figure 4:**
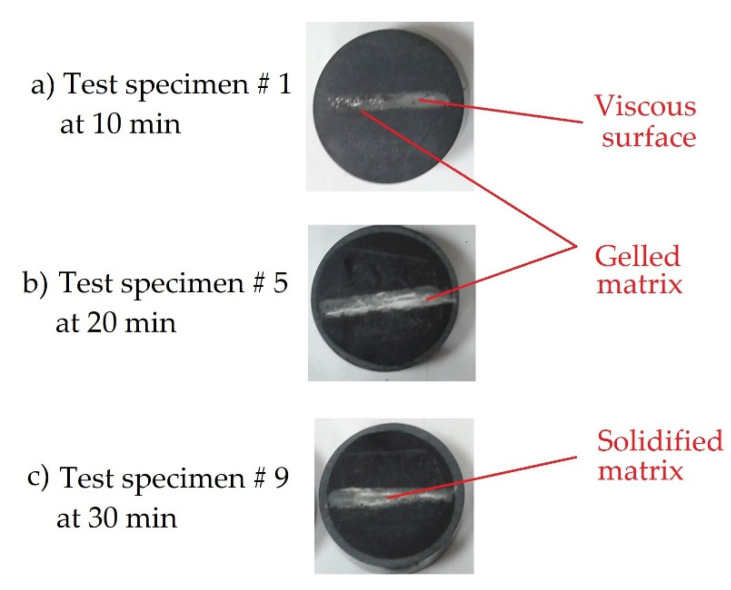
Stuffed material specimens with curing times of (**a**) 10, (**b**) 20 and (**c**) 30 min respectively.

The sandpaper dust used in polishing the exposed sections in the inlaid materials allowed us to achieve a good finish on the observation surface. This is observed in Figure 4, in which the samples are presented at the different time intervals. 

The capsules are cylindrical, and the sections clearly have the layers of the cured prepreg panels identified. In Figure 4a of 10 min, in Figure 4b of 20 min and in Figure 4c of 30 min. Test specimen # 1 shown in Figure 4a was still viscous to the touch as well as in gel form. In Figure 4b, corresponding to test specimen # 5, gelled areas were observed after cleaning the sandpaper, which could no longer be easily removed. In Figure 4c corresponding to test specimen # 9, the matrix was completely solid and there were no more gelled parts in the laminate. All this was corroborated under the microscope.

**Figure 5:**
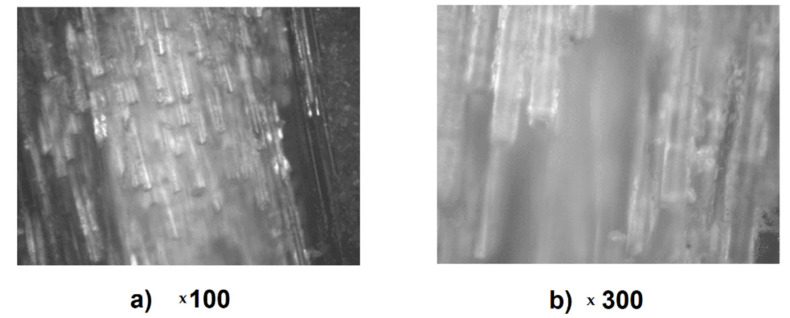
Stuffed material specimens with cure times of 10 min at zoom of (**a**) ×100 and (**b**) ×300.

In the case of stuffed materials observed under ×100 zoom in the microscope (Center for Research in Structural Materials–CIME, Madrid, Spain) as seen in Figure 5, there is liquid resin between the fibers. Some fibers even have the appearance of being wet. The resin has not bonded the layers, but no gas porosities are observed that indicate that they will be trapped in the matrix or between the layers. The ×300 zoom presented in Figure 5b in the same Figure 5, allows us to observe a lake of liquid resin wetting the layers of the prepreg. At this level increases, no porosities are observed. The laminate is cohesive and has good adhesion.

**Figure 6:**
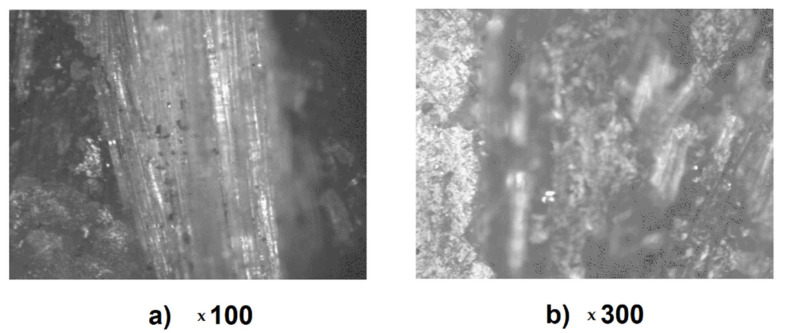
Stuffed material specimens with cure times of 20 min at zoom of (**a**) ×100 and (**b**) ×300.

The specimens with a curing time of 20 min at 120 degrees C are shown in Figure 6. At ×100 zoom, fibers are already cohesive with the resin, and the matrix has even adopted an opaque color, displaying small areas with suspended solids, that is, the monomers of the matrix have already finished nucleating between the fibers. At ×300 zoom it is easier to see the already solidified areas, although at this zoom level some of the matrix is still visible in gel form. They are no longer any large liquid stains, but rather intermixed with solids, which shows good cohesion between the matrix and the fiber. The laminate is observed to be already cohesive.

**Figure 7:**
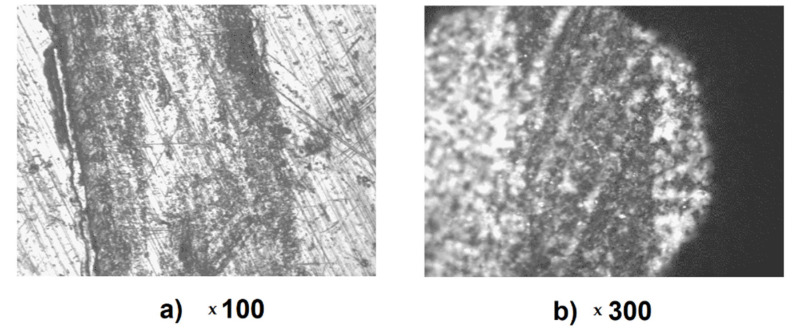
Stuffed material specimens with cure times of 30 min at zoom of (**a**) ×100 and (**b**) ×300.

The specimens of stuffed material with a curing time of 30 min at 120 degrees C are observed under ×100 zoom in Figure 7a where they are solidified. There is a very clear difference between the fiber and the matrix with the orientation of the layers. The curing process is almost complete. In the same Figure 7b, it can be seen at ×300 zoom that there are no longer any matrix gap gelling, and everything is solid. The fiber orientations are shown as color differences for each of the layers and there are a lot of clearly defined particles. Spherical porosities are observed, indicating the presence of gases trapped in the curing process.

### 3.2. Tests for Vertical Weight Drop and Exposure to Ultraviolet Light

The panels impacted with energies of 10, 20, 30 and 40 Joules, which is a reference value to associate with the results, were characterized by penetrating fluorescent inks. The impacts correspond to those presented in Table 2. It shows the impacts of four panels which were tested with a weight of 5.549 kg released at different heights. These energies applied to the panels have been presented in a referential way at their 9.97/20.27/30.09/39.37 Joules. The equipment has an anti-bounce sensor in such a way that they were tested with a single impact. The anti-bounce system is equipped with a laser sensor and guaranteed that this condition is fulfilled satisfactorily.

**Figure 8:**
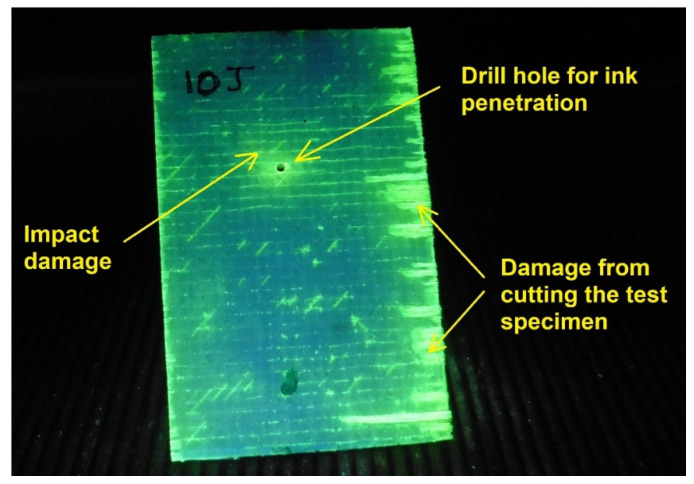
Specimen tested at 10 joules impact after being immersed in penetrant ink and exposed to ultraviolet light.

In Figure 8, a specimen tested at 10 Joules of impact is shown with its central portion removed from the entire panel. This is for the purpose of working with the fluorescent penetrating ink only in the damaged area. In it we can see the hole that was drilled so that when the test tube is immersed in the ink, it penetrates. It should be noted that the drilling was carried out at the point of impact in order to minimize the damage caused by this action. Lateral damage is observed in the test tube, which is due to saw cutting, in which the ink penetrated, but without damaging the impact area.

**Figure 9:**
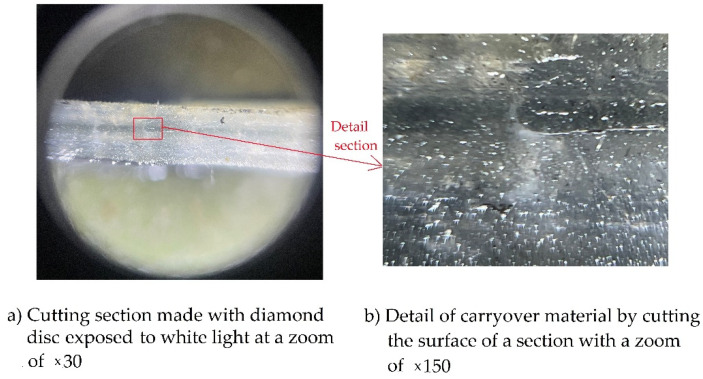
Observation of a section hits at 10 Joules (**a**) ×30 zoom, (**b**) ×150 zoom.

Using a magnifying lens with white light, the cut sections were observed for the purpose of evaluating the drag material of the diamond cut. Figure 9a shows the cross-section of a specimen impacted at 10 Joules. The brightness of the white light allows it to reflect the variations in the surface caused by incrustations or discontinuities in which shadows or highlights are reflected to be qualitatively evaluated. It is interesting that white light also makes it possible to identify the delamination between layers of the composite material due to the change of direction of the light on the surface. Also, the porosities that remained due to the effect of the gases during the curing process are observable as shadows on the surface. In general, the surface is well cohesive between the matrix of each layer and this is in accordance with the tests of the curing time of the material which indicated that the matrix and the fiber were well adhered to each other.

The microscope equipment allows a zoom ×5.5 times the initial one, and this effectively allows observing that despite the use of lubricating oil during diamond cutting, particles of matrix and fiber material were dragged and adhered to the surface of the section exposed to white light according to Figure 9b. The type of particle adhered and its distribution on the surface will however not influence the results. The particles are oriented in a circular way and this is due to the fact that the blade is a circular disk, and it is pressing them on the composite material.

**Figure 10:**
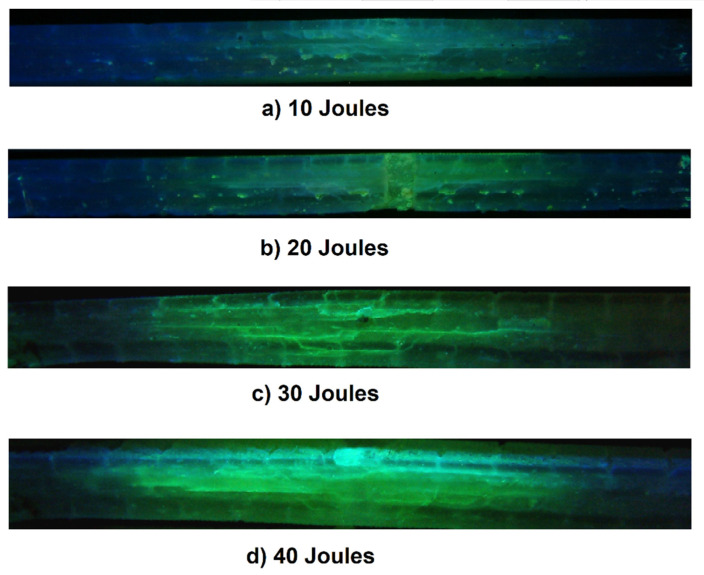
Characterization with ultraviolet light to the sections of the laminates in the impact zone, (**a**) 10 joules, (**b**) 20 joules, (**c**) 30 joules and (**d**) 40 joules.

All specimens were cut according to the experimental method to expose the impacted cross-sections. The oil used with the diamond blade cut effectively prevented matrix particles from entraining into the delamination, pores, or breaks in the resin. Figure 10 shows these sections exposed to ultraviolet light. The damage increases as the impact energy is increased, allowing us to see the different breaks, cracks and staggering effects formed as the impactor damages the laminate. There is a very clearly delimited delamination, as well as easily observable matrix breaks.

**Figure 11:**
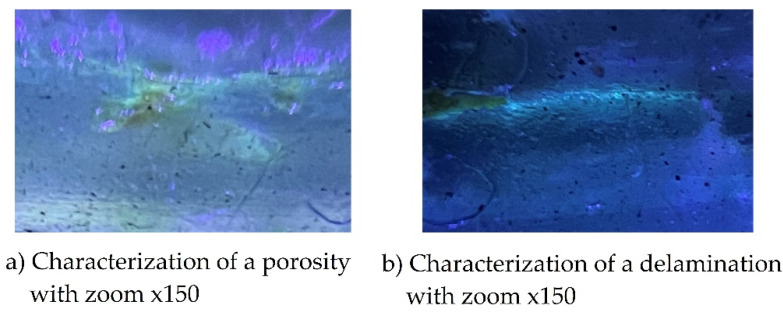
Characterization with ultraviolet light to a section impacted with 20 Joules with (**a**) porosity viewed at ×150 zoom, (**b**) delamination at ×150 zoom.

To evaluate the failures in the matrix, the cut sections were observed with an increase of ×150 as observed in Figure 11a. There is porosity in the circular shaped matrix which indicates that gases were trapped during the curing process. The quantity of pores evaluated qualitatively is not very significant, but it does influence the results. In Figure 11 it is observed that the rupture of the matrix is around two larger pores in relation to the others exposed in the image. The penetrating ink has painted the surrounding area allowing concentration and stresses and damaging the matrix. There are also areas with pores in which there has been no damage to the matrix. The different shades of the green color of the fluorescent penetrating ink is an indicator of the magnitude of the damage, because the more accentuated the green color, the greater the damage in the laminate layer. It could not be identified by the type of increase which is the layer that corresponds to this damage, but it is estimated that it is between number 2 and number 3 of the first prepreg fabric composed of three warps. Figure 11b shows a delamination identified by the color of the fluorescent penetrating ink for an impact at 20 Joules magnified ×150. There are several pores on the delamination zone, but these do not influence the laminate damage. The ultraviolet light shows that the ink has penetrated between the layers, indicating a cohesion failure caused by the impact. No cracks in the matrix or microcracks are observed. This section shown corresponds to layers 1 and 2 of the first warp. It should be noted that in Figure 11b an entire area shaded with fluorescent ink is observed, and from the image obtained at ×150 zoom is ruled out that it is a surface with a large number of microcracks since it is clean of damage and the defects or porosities have not affected the matrix core or produced any damage.

**Figure 12:**
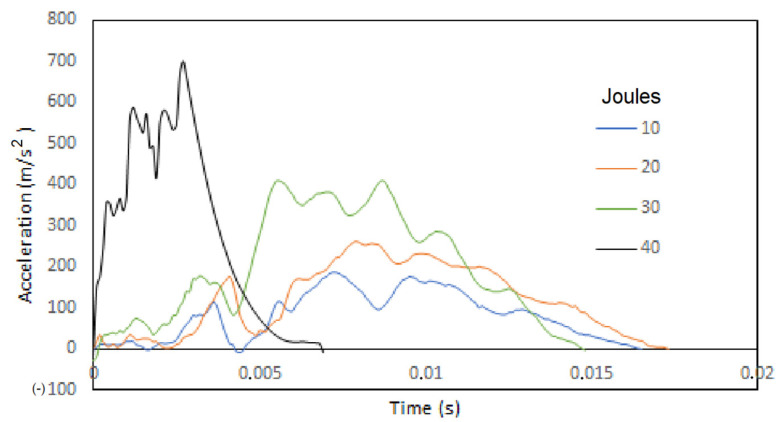
Acceleration profile (m/s^2^) of the panels during deformation for impacts of 10, 20, 30 and 40 joules.

The gravitometer with a frequency of 4 × 10^3^ Hz tested the accelerations of the de-formations of the panels during the impact. That is, it registered accelerations and decelerations while the layers suffered intralaminar and interlaminar damage. The acceleration profile for the 10, 20, 30 and 40 Joules tests are shown in Figure 12. The results are acceptable because in the first instance there is an increase in acceleration as the impact energy increases and it is clearly due to the fact that with increasing weight, the potential energy transferred when the impactor touches the surface of the panel is greater. Additionally, the effective recording times that are used for the results, decrease with higher energy and this is due to the deformation speed. The delamination produces continuities in the acceleration lines due to the tension in the adhesion between layers since the equipment tests the detachment of the layers that is taking place, while the rupture of the matrix produces peaks or jumps in the records.

**Figure 13:**
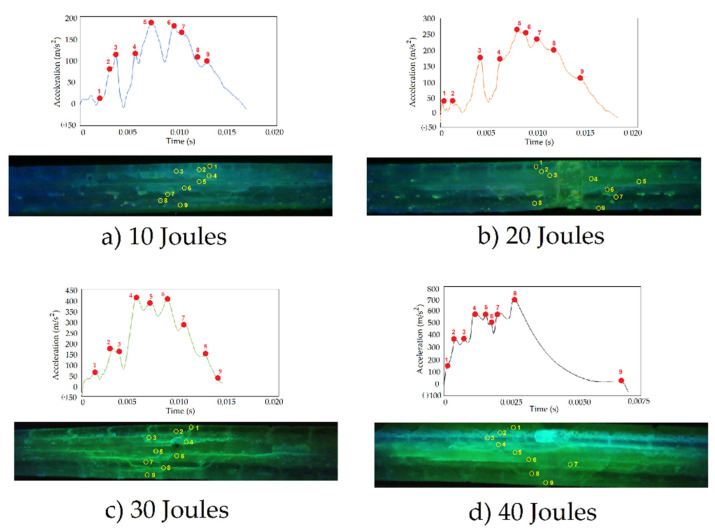
Analysis of the acceleration (m/s^2^) and its relationship with the damage presented in the cross section using fluorescent light at (**a**) 10 joules, (**b**) 20 Joules, (**c**) 30 Joules and (**d**) 40 Joules.

When the impact on the laminate occurs at 10 joules, the configuration of the laminate due to its orthotropic means that the greatest damages tested by the gravitometer are oriented over the same area, showing a kind of continuity as seen in Figure 13a. The rupture of the matrix (observation given by the type of spots under the fluorescent light of this area without horizontal lines) follows the orientation of the layers at the indicated points 1, 2 and 3 which corresponds to a factory prepreg sheet. For this reason, a jump is observed in the rupture of the matrix from layer 3 to 4, in which the adhesion of the fabrics is acting, which was observed in the curing time and which was acceptable. The group of sheets 4, 5 and 6 are broken mainly following the same trend of the previous prepreg group. In the case of sheets 7, 8 and 9 there is a slight difference in the behavior of the break, noting that layer 9 has a large extent of break in the matrix because it is the one with the greatest deflection.

**Figure 14:**
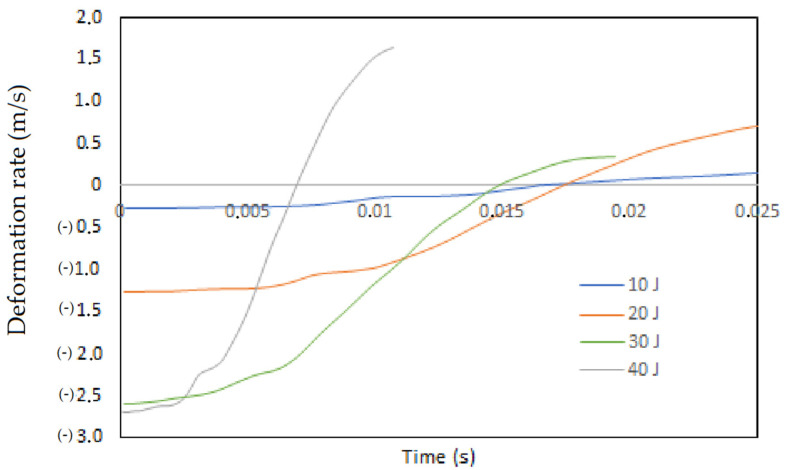
Analysis of the deformation speed (m/s) of the panels tested at 10, 20, 30 and 40 Joules.

When the impact on the selected laminate occurs with an energy of 20 Joules, the orthotropy of the material plays a different role than with 10 Joules. This is seen in Figure 13b. Adhesion produces a greater variation in the micro-failure cracks in the matrix. In the first sheet of three layers of prepreg, a higher breaking tendency is maintained in a zone close to each other, indicating that the cohesion between layers remains strong. This effect corresponds to points 1, 2 and 3 in which there are no greater acceleration peaks. Between 3 and 4 appears the effect of the curing of the prepreg sheets, therefore a jump in acceleration is observed. The impactor tends to slow down, but as the layer yields, it increases its acceleration again until layer 4 breaks. When the laminate is deformed on the fourth layer that corresponds to the second prepreg fabric there is a noticeable difference in the location of the tear of the matrix and this causes the areas of greatest breakage to separate within the laminate producing greater damage. We see this at points 4, 5 and 6. This effect is very different between layer 6 and 7 because the impacted one must break a greater thickness of the laminate. The third prepreg fabric corresponding to layers 7, 8 and 9 have a certain uniformity and tend to show their greatest tear in a very close area. It is the area of greatest flexion and the impactor is decelerating until restitution is achieved. According to what is observed in Figure 14, the surfaces painted with fluorescent ink do not correspond to generalized microcracks, but rather to stains produced by the characterization.

In the case of the 30 Joules test, the evaluation of the points of greatest breakage is more difficult due to the appearance of staggered delaminations that join the matrix with the adhesion of the layers. This is seen in Figure 13c. In the first prepreg fabric manufactured with three layers, the damage corresponds exclusively to the matrix for layer 1 and 2. From layer 2 to 3, there is significant delamination despite being of the same fabric as the first group. This joins with the rupture of the previous matrix, giving it continuity. In the case of the next group of three layers, the same effect is produced. On layer 6 there is a significant delamination with the difference that in this group the vertical breaks of the matrix are much smaller in quantity, and those that have occurred have been staggered with the previous delamination. Here, between 6 and 7, adhesion failure occurred, acting on the cohesion force between the fabrics as a result of the deflection of the panel during impact. For the last group of laminated fabric, the breaks are minor. Between layers 7 and 8 there is a significant delamination in the form of a step.

With the impact at 40 Joules, it is observed that the matrix presents considerable damage in terms of microcracks and generalized breaks. As can be seen in Figure 13d, the staggering corresponding to the delamination between layers fades with the spots of generalized damage in the matrix. In the first fabric with 3 layers of prepreg, it is observed that in layers 1 and 2 there is little fade damage and layer 3, due to the orthotopic nature of the material, presents vertical breaks in the matrix. The union of the first group of pre-impregnated fabric with the second one exhibits a significant delamination that spreads to the following layers of the laminate. Layers 4, 5 and 6 of this group of fabrics are totally damaged and the matrix is completely broken. This corresponds to layers 4, 5 and 6. Between the second and third prepreg fabrics, we observe that the damage decreases and occurs mainly in the form of delamination with vertical breaks of the matrix in a staggered manner. At this level of impact, the layer 9 has been broken in a greater section by the greater bending during the deformation. Due to the level of damage in the central layers, it is observed that there was good adhesion between prepreg fabrics, resisting cohesion very well during curing.

The curves obtained by applying Equation (1) to present the behavior of the impactor speed at which the impacted panels are deformed and restored at 10, 20, 30 and 40 Joules, are shown in Figure 14. Effectively, as the energy-imposed deformation will be higher, the results show that the impact at 10 Joules has a shorter time than the impact at 20 Joules, which is directly related to the selected 4×10^3^ Hz frequency. A greater frequency range would have presented a greater convergence in the results. Above 30 Joules of impact, there is a notable change in the deformation and restitution speed of the specimen when it is impacted, indicating that the tests become high-energy impacts and therefore require a different treatment in the handling of the results. Additionally, in the curves, it is observed that orthotropy of the layers is present as a discontinuity in the curve after the onset of the impact.

**Figure 15:**
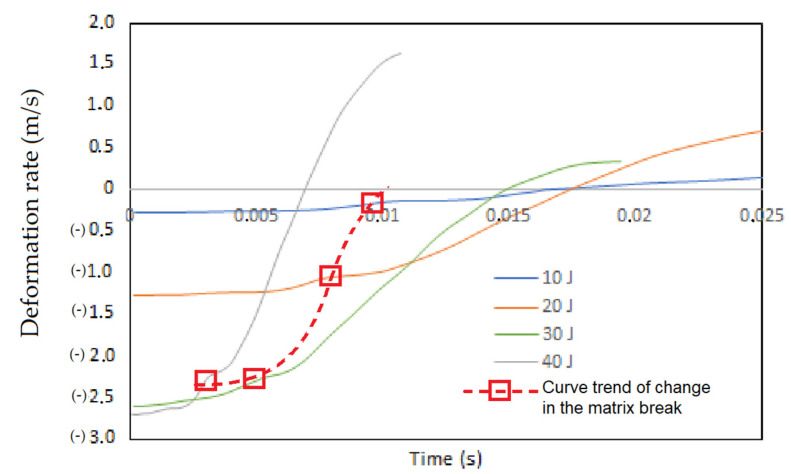
Definition of the trend curve that produces changes in the ability to resist the rupture of the matrix for deformation speeds (m/s) at impact energies at 10, 20, 30 and 40 Joules.

In accordance with the trend, the variation that produces a change in the behavior of the matrix and produces greater damage is fully identified. In Figure 15 this curve is formed by the union of discontinuous data in the different tests showing a behavior that tends to be horizontal, that is, the quantification of damage in a qualitative way will not show a great variation at the beginning of the impact for values greater than 30 Joules. in the case of the laminate presented. On the other hand, for tests of less than 30 Joules, the tendency for the matrix to break has a quasi-linear behavior according to how the first intralaminar and interlaminar damages appear at the beginning of the impact. As there are no other discontinuous points, no other trend curves can be established within the observed results.

## 4. Discussion

The useful life of a GFRP laminate is determined based on knowing how it responds to different working circumstances such as deformation, failure due to adherence and concentrated loads that cause breakage. The energy applied, depending on its magnitude, can be related to punctual phenomena or repetitive fatigue events. In such a way, establishing trends in the behavior of a GFRP laminate to be installed in a structure is a way of designing and dtermining its limitations and manufacturing details.

With the results presented, the question is: Is it possible with this process to understand how a GFRP laminated panel will respond to the conditions of the work for which it was designed? The answer is “yes”. In the first instance, the curing control processes must be respected and, if possible, the laminate should be sampled once it has been produced.

The observation of the curing at a maximum of 30 min gave a good representation that the manufacturing process of the panels at the full time of preparation is sufficient to have a very well cohesive laminate, and that it allows good results to be obtained in the subsequent experiments. At 30 min, the prepreg was solidified to a high percentage close to 100%, in such a way that it can be guaranteed the delamination and breaks observed in the tests of exposure to fluorescent penetrating ink are effectively damage from impacts and not damage resulting from its manufacturing. This was verified qualitatively with the ×150 observation since indeed there were pores caused by gases trapped during the curing but that influenced the results.

To relate all the results, it is essential that all the peaks in which the delamination occur are evaluated in the acceleration graph. As can be seen in the results, this allows us to analyze its influence on the orientation of the layers. The laminate selections showed that its stacking configuration always had the same tendency to present peaks between the same layers, independent of the impact energy load. This is what allows establishing the trend of the “laminate damage threshold”, that is, when the observer indicates that the laminate is not capable of working and will have structural failure.

The observation of the impacted sections allows one to have a clear idea of the values at which the damage tendency of a selected laminate will change. In the results of the present study, there is clearly a change in the trend for the selected laminate configuration between 30 and 40 Joules. This means that with this study it is clearly established that the configuration will not be recommended to work within these limits. This includes fatigue, as the accumulation of damage equates to the accumulation of energy per impact.

The acceleration and velocity graphs are very clear in presenting this result and leave no other alternative for the use of the selected GFRP. It should be noted that the observation did not require sophisticated microscopes or high-level microscopy, but rather a rational magnification level attainable with equipment accessible to most researchers is sufficient to know the influence of porosities and imperfections of the laminate. In the presented study presented these imperfections, do not affect the results.

## 5. Conclusions

The proposed methodology makes it possible to establish for a particular laminate configuration, its tendency to resist impact due to its layer orientation, establishing the energy values at which the GFRP loses its response capacity. As observed in Figure 15, the panel responds well to the energy applied, returning it during restitution, which is confirmed by the shape of the velocity curve, but it completely loses its ability to return the damage at values greater than 30 Joules. This means that its useful life, its use in the construction of structures, boats, vehicles and others will not meet its objective for these values. In such a way that the methodology is applied to other configurations of laminates and composite materials, researchers can establish the maximum values of work and design in a qualitative way.

The evaluation of the behavior of a laminate configuration to damage by energy impacts, as well as qualitatively quantifying the interlaminar and intralaminar delamination that occur, clearly allows us to know how the GFRP will respond when it is in use. It means that researchers can use this methodology to adapt it to their investigations for the purpose of establishing the life of a laminate.

## Figures and Tables

**Table 1:** Result of the curing processes of the test specimens for embedding and microscopic observation.

Test Specimen #	Time (min)	Observation
1	10	The procedure was adequate, it was turned off 10 s later
2	10	The specimen was adequate in time and temperature
3	10	The specimen was adequate in time and temperature
4	20	The oven failed 5 min before the expected time
5	20	The procedure was adequate, it was turned off 30 s later
6	20	The specimen was adequate in time and temperature
7	30	The specimen was adequate in time and temperature
8	30	The specimen was adequate in time and temperature
9	30	The procedure was adequate, it was turned off 5 s before curing time

**Table 2:** Impact energies (J) applied to the prepreg panels by vertical drop in weight.

Panel	Weight Drop (kg)	Drop Height (cm)	Impact Energy (J)	Reference Value (J)
1	5.549	18.31	9.97	10
2	5.549	37.23	20.27	20
3	5.549	55.28	30.09	30
4	5.549	73.24	39.87	40

## Data Availability

Data sharing not applicable.
